# Supplementary material for: Are dietary factors associated with cardiometabolic risk factors in patients with non-alcoholic fatty liver disease?
Source: PeerJ. 2024 Jul 30;12:e17810. doi: 10.7717/peerj.17810 (PMC11296304; doi:10.7717/peerj.17810)
Supplement: Supplemental Information 3 — ANOVA (Tukey’s). Kruskal Wallis. chi-square. Bonferroni adjustment. Bold indicates statistically significant difference (p ≤ 0.05). Results with different letters (a-d) in the same row are significantly different. Cut-off points for men; DII-Q1: 16.10-34.41, DII-Q2: 34.89-40.46, DII-Q3: 42.25-49.55, DII-Q4: 49.76-69.00; DIL-Q1: 12395.39-130971.78, DIL-Q2: 171779.47-257751.76, DIL-Q3: 260287.76-349267.01, DIL-Q4: 374115.85-702706.49, DGI-Q1: 29.10-46.98, DGI-Q2: 50.05-56.01, DGI-Q3: 57.33-63.75, DGI-Q4: 64.06-251.40; DGL-Q1: 12.40-52.39, DGL-Q2: 60.93-81.96, DGL-Q3: 88.78-135.78, DGL-Q4: 138.57-608.58. Cut-off points for women; DII-Q1: 16.05-34.73, DII-Q2: 35.42-41.65, DII-Q3: 41.86-49.13, DII-Q4: 49.70-69.71; DIL-Q1: 49517.53-155328.38, DIL-Q2: 163285.85-254563.25, DIL-Q3: 259840.92-362815.22, DIL-Q4: 398178.02-784256.20, DGI-Q1: 28.55-46.71, DGI-Q2: 48.68-57.25, DGI-Q3: 57.36-63.34, DGI-Q4: 65.36-252.63; DGL-Q1: 11.62-54.47, DGL-Q2: 56.93-85.36, DGL-Q3: 86.69-134.35, DGL-Q4: 138.08-252.74. [file peerj-12-17810-s003.docx]

**Supplementary Table 1.** Demographic characteristics of the participants in each quartile of DII, DIL, DGI, and DGL.

|  | ***DII*** | | | | | ***DIL*** | | | | | ***DGI*** | | | | | | ***DGL*** | | | | |
| --- | --- | --- | --- | --- | --- | --- | --- | --- | --- | --- | --- | --- | --- | --- | --- | --- | --- | --- | --- | --- | --- |
|  | **Q1** | **Q2** | **Q3** | **Q4** | **p** | **Q1** | **Q2** | **Q3** | **Q4** | **p** | **Q1** | **Q2** | **Q3** | **Q4** | **p** | **Q1** | | **Q2** | **Q3** | **Q4** | **p** |
| **Men** | | | | | | | | | | | | | | | | | | | | | |
| **Frequency, n** | 14 (25.0) | 12 (21.4) | 13 (23.2) | 17 (30.4) |  | 12 (21.4) | 9 (16.1) | 14 (25.0) | 21 (37.5) |  | 10 (17.9) | 11 (19.6) | 16 (28.6) | 19 (33.9) |  | 8 (14.3) | | 12 (21.4) | 13 (23.2) | 23 (41.1) |  |
| **Age** | 40.6±10.40 | 37.2±12.04 | 41.5±13.07 | 40.9±12.04 | 0.795 | 42.0±10.37 | 36.9±11.72 | 40.2±14.24 | 40.6±11.09 | 0.804 | 39.6±13.20 | 36.8±10.77 | 41.7±9.28 | 41.2±13.57 | 0.729 | 42.3±10.42 | | 40.3±12.67 | 39.1±12.86 | 40.0±11.60 | 0.949 |
| **Physical Activity Level (IPAQ)** | | | | | | | | | | | | | | | | | | | | | |
| ***Inactive*** | 4 (7.1) | 8 (14.3) | 7 (12.5) | 12 (21.4) | 0.098 | 8 (14.3) | 5 (8.9) | 6 (10.7) | 12 (21.4) | 0.675 | 5 (8.9) | 5 (8.9) | 9 (16.1) | 12 (21.4) | 0.795 | 9 (16.1) | | 7 (12.5) | 8 (14.3) | 13 (23.2) | 0.732 |
| ***Minimal Active*** | 10 (17.9) | 4 (7.1) | 6 (10.7) | 5 (8.9) |  | 4 (7.1) | 4 (7.1) | 8 (14.3) | 9 (16.1) |  | 5 (8.9) | 6 (10.7) | 7 (12.5) | 7 (12.5) |  | 5 (8.9) | | 5 (8.9) | 5 (8.9) | 10 (17.9) |  |
| ***Active*** | 0 (0) | 0 (0) | 0 (0) | 0 (0) |  | 0 (0) | 0 (0) | 0 (0) | 0 (0) |  | 0 (0) | 0 (0) | 0 (0) | 0 (0) |  | 0 (0) | | 0 (0) | 0 (0) | 0 (0) |  |
| **Waist circumference (Normal-Above; ≥94 cm for men. and ≥80 cm for women)** | | | | | | | | | | | | | | | | | | | | | |
| ***Normal*** | 5 (8.9) | 2 (3.6) | 2 (3.6) | 3 (5.4) | 0.515 | 2 (3.6) | 3 (5.4) | 2 (3.6) | 5 (8.9) | 0.702 | 1 (1.8) | 2 (3.6) | 2 (3.6) | 7 (12.5) | 0.232 | 1 (1.8) | | 3 (5.4) | 2 (3.6) | 6 (10.7) | 0.790 |
| ***Above*** | 9 (16.1) | 10 (17.9) | 11 (19.6) | 14 (25.0) |  | 10 (17.9) | 6 (10.7) | 12 (21.4) | 16 (28.6) |  | 9 (16.1) | 9 (16.1) | 14 (25.0) | 12 (21.4) |  | 7 (12.5) | | 9 (16.1) | 11 (19.6) | 17 (30.4) |  |
| **Body fat percentage (%)** | 27.8±7.69 | 25.5±5.28 | 29.2±8.56 | 27.0±6.05 | 0.623 | **31.79±9.25^a^** | **23.59±4.45^b^** | **27.79±4.97^ab^** | **26.49±6.45^ab^** | **0.035** | 31.9±5.97 | 25.5±6.70 | 27.0±6.73 | 26.5±7.12 | 0.135 | 29.1±6.51 | | 25.5±5.85 | 29.3±7.19 | 26.7±7.43 | 0.479 |
| **Body Mass Index-BMI (kg/m^2^)** | 31.3±6.68 | 29.3±3.61 | 29.5±4.30 | 29.8±4.55 | 0.696 | 33.4±5.90 | 28.1±3.91 | 29.1±3.26 | 29.4±4.90 | 0.059 | 33.4±4.80 | 28.9±4.89 | 28.8±3.62 | 29.9±5.37 | 0.092 | 32.4±4.57 | | 28.6±3.65 | 30.5±4.64 | 29.6±5.59 | 0.380 |
| **Body Mass Index-BMI (kg/m^2^)** | | | | | | | | | | | | | | | | | | | | | |
| ***25.0-29.9 kg/m^2^*** | 7 (12.5) | 8 (14.3) | 5 (8.9) | 9 (16.1) | 0.343 | 3 (5.4) | 6 (10.7) | 10 (17.9) | 10 (17.9) | 0.219 | 2 (3.6) | 7 (12.5) | 10 (17.9) | 10 (17.9) | 0.474 | 3 (5.4) | | 9 (16.1) | 6 (10.7) | 11 (19.6) | 0.478 |
| ***30.0-34.9 kg/m^2^*** | 4 (7.1) | 3 (5.4) | 8 (14.3) | 6 (10.7) |  | 6 (10.7) | 3 (5.4) | 3 (5.4) | 9 (16.1) |  | 6 (10.7) | 3 (5.4) | 5 (8.9) | 7 (12.5) |  | 3 (5.4) | | 3 (5.4) | 6 (10.7) | 9 (16.1) |  |
| ***≥35.0 kg/m^2^*** | 3 (5.4) | 1 (1.8) | 0 (0) | 2 (3.6) |  | 3 (5.4) | 0 (0) | 1 (1.8) | 2 (3.6) |  | 2 (3.6) | 1 (1.8) | 1 (1.8) | 2 (3.6) |  | 2 (3.6) | | 0 (0) | 1 (1.8) | 3 (5.4) |  |
|  | ***DII*** | | | | | ***DIL*** | | | | | ***DGI*** | | | | | | ***DGL*** | | | | |
|  | **Q1** | **Q2** | **Q3** | **Q4** | **p** | **Q1** | **Q2** | **Q3** | **Q4** | **p** | **Q1** | **Q2** | **Q3** | **Q4** | **p** | **Q1** | | **Q2** | **Q3** | **Q4** | **p** |
| **Women** | | | | | | | | | | | | | | | | | | | | | |
| **Frequency, n** | 15 (24.6) | 18 (29.5) | 16 (26.2) | 12 (19.7) |  | 17 (27.9) | 20 (32.8) | 16 (26.2) | 8 (13.1) |  | 19 (31.1) | 19 (31.1) | 13 (21.3) | 10 (16.4) |  | 21 (34.4) | | 18 (29.5) | 16 (26.2) | 6 (9.8) |  |
| **Age** | 50.7±7.24 | 50.3±7.59 | 51.6±9.87 | 44.6±11.58 | 0.200 | 50.5±8.23 | 50.8±8.33 | 50.7±10.85 | 42.6±7.74 | 0.147 | 50.4±8.60 | 48.4±10.18 | 49.7±9.29 | 50.3±9.23 | 0.921 | 50.2±9.48 | | 52.3±6.50 | 47.8±11.01 | 44.2±8.13 | 0.217 |
| **Physical Activity Level (IPAQ)** | | | | | | | | | | | | | | | | | | | | | |
| ***Inactive*** | 11 (18.0) | 14 (23.0) | 14 (23.0) | 10 (16.4) | 0.770 | 11 (18.0) | 17 (27.9) | 14 (23.0) | 7 (11.5) | 0.298 | 16 (26.2) | 16 (26.2) | 10 (16.4) | 7 (11.5) | 0.769 | 17 (27.9) | | 15 (24.6) | 12 (19.7) | 5 (8.2) | 0.934 |
| ***Minimal Active*** | 4 (6.6) | 4 (6.6) | 2 (3.3) | 2 (3.3) |  | 6 (9.8) | 3 (4.9) | 2 (3.3) | 1 (1.6) |  | 3 (4.9) | 3 (4.9) | 3 (4.9) | 3 (4.9) |  | 4 (6.6) | | 3 (4.9) | 4 (6.6) | 1 (1.6) |  |
| ***Active*** | 0 (0) | 0 (0) | 0 (0) | 0 (0) |  | 0 (0) | 0 (0) | 0 (0) | 0 (0) |  | 0 (0) | 0 (0) | 0 (0) | 0 (0) |  | 0 (0) | | 0 (0) | 0 (0) | 0 (0) |  |
| **Waist circumference (Normal-Above; ≥94 cm for men. and ≥80 cm for women)** | | | | | | | | | | | | | | | | | | | | | |
| ***Normal*** | 0 (0) | 0 (0) | 0 (0) | 0 (0) | 0.999 | 0 (0) | 0 (0) | 0 (0) | 0 (0) | 0.999 | 0 (0) | 0 (0) | 0 (0) | 0 (0) | 0.999 | 0 (0) | | 0 (0) | 0 (0) | 0 (0) | 0.999 |
| ***Above*** | 15 (24.6) | 18 (29.5) | 16 (26.2) | 12 (19.7) |  | 17 (27.9) | 20 (32.8) | 16 (26.2) | 8 (13.1) |  | 19 (31.1) | 19 (31.1) | 13 (21.3) | 10 (16.4) |  | 21 (34.4) | | 18 (29.5) | 16 (26.2) | 6 (9.8) |  |
| **Body fat percentage (%)** | 37.9±7.09 | 38.1±5.87 | 38.9±5.16 | 39.2±4.72 | 0.924 | 36.19±6.93 | 39.59±5.30 | 38.79±5.09 | 40.49±4.01 | 0.212 | 39.4±4.34 | 39.0±5.80 | 38.5±4.11 | 35.7±8.86 | 0.406 | 38.3±6.51 | | 35.5±7.68 | 37.6±4.74 | 41.3±3.64 | 0.612 |
| **Body Mass Index-BMI (kg/m^2^)** | 32.6±6.01 | 33.1±6.26 | 33.5±4.56 | 34.7±5.25 | 0.804 | 31.7±6.29 | 34.1±5.01 | 32.7±5.37 | 36.7±4.24 | 0.170 | 33.4±5.63 | 33.1±5.06 | 33.8±5.31 | 33.5±7.04 | 0.987 | 32.4±5.02 | | 32.5±6.07 | 34.5±5.79 | 36.8±3.44 | 0.243 |
| **Body Mass Index-BMI (kg/m^2^)** | | | | | | | | | | | | | | | | | | | | | |
| ***25.0-29.9 kg/m^2^*** | 6 (9.8) | 6 (9.8) | 3 (4.9) | 3 (4.9) | 0.693 | 7 (11.5) | 6 (9.8) | 5 (8.2) | 0 (0) | 0.167 | 3 (4.9) | 7 (11.5) | 4 (6.6) | 4 (6.6) | 0.219 | 6 (9.8) | | 8 (13.1) | 4 (6.6) | 0 (0) | 0.195 |
| ***30.0-34.9 kg/m^2^*** | 5 (8.2) | 7 (11.5) | 5 (8.2) | 3 (4.9) |  | 6 (9.8) | 5 (8.2) | 7 (11.5) | 2 (3.3) |  | 11 (18.0) | 4 (6.6) | 3 (4.9) | 2 (3.3) |  | 8 (13.1) | | 6 (9.8) | 5 (8.2) | 1 (1.6) |  |
| ***≥35.0 kg/m^2^*** | 4 (6.6) | 5 (8.2) | 8 (13.1) | 9 (14.8) |  | 4 (6.6) | 9 (14.8) | 4 (6.6) | 6 (9.8) |  | 5 (8.2) | 8 (13.1) | 6 (9.8) | 4 (6.6) |  | 7 (11.5) | | 4 (6.6) | 7 (11.5) | 5 (8.2) |  |

ANOVA (Tukey’s). Kruskal Wallis. chi-square. Bonferroni adjustment.

Bold indicates statistically significant difference (*P*≤0.05). Results with different letters (a-d) in the same row are significantly different.

Cut-off points for men; DII-Q1: 16.10-34.41, DII-Q2: 34.89-40.46, DII-Q3: 42.25-49.55, DII-Q4: 49.76-69.00; DIL-Q1: 12395.39-130971.78, DIL-Q2: 171779.47-257751.76, DIL-Q3: 260287.76-349267.01, DIL-Q4: 374115.85-702706.49, DGI-Q1: 29.10-46.98, DGI-Q2: 50.05-56.01, DGI-Q3: 57.33-63.75, DGI-Q4: 64.06-251.40; DGL-Q1: 12.40-52.39, DGL-Q2: 60.93-81.96, DGL-Q3: 88.78-135.78, DGL-Q4: 138.57-608.58.

Cut-off points for women; DII-Q1: 16.05-34.73, DII-Q2: 35.42-41.65, DII-Q3: 41.86-49.13, DII-Q4: 49.70-69.71; DIL-Q1: 49517.53-155328.38, DIL-Q2: 163285.85-254563.25, DIL-Q3: 259840.92-362815.22, DIL-Q4: 398178.02-784256.20, DGI-Q1: 28.55-46.71, DGI-Q2: 48.68-57.25, DGI-Q3: 57.36-63.34, DGI-Q4: 65.36-252.63; DGL-Q1: 11.62-54.47, DGL-Q2: 56.93-85.36, DGL-Q3: 86.69-134.35, DGL-Q4: 138.08-252.74.
