# Supplementary material for: Are dietary factors associated with cardiometabolic risk factors in patients with non-alcoholic fatty liver disease?
Source: PeerJ. 2024 Jul 30;12:e17810. doi: 10.7717/peerj.17810 (PMC11296304; doi:10.7717/peerj.17810)
Supplement: Supplemental Information 4 — ANOVA, Kruskal Wallis, Chi-square [file peerj-12-17810-s004.docx]

**Supplementary Table 2.** Biochemical parameters of the participants in each quartile of DII and DIL.

|  | ***DII*** | | | | | ***DIL*** | | | | | |
| --- | --- | --- | --- | --- | --- | --- | --- | --- | --- | --- | --- |
|  | **Q1** | **Q2** | **Q3** | **Q4** | **p** | **Q1** | **Q2** | **Q3** | **Q4** | **p** |  |
| **Men** | | | | | | | | | | |  |
| **ALT (alanine transaminase)** | 50.2±25.96 | 74.8±38.23 | 64.7±72.00 | 56.2±21.31 | 0.487 | 59.6±18.85 | 45.8±24.78 | 45.3±19.72 | 78.0±60.55 | 0.088 |  |
| **AST (aspartate transaminase)** | 31.5±9.44 | 42.7±21.29 | 36.5±24.84 | 32.8±9.85 | 0.348 | 35.1±12.00 | 29.1±9.58 | 31.4±8.82 | 41.0±23.99 | 0.241 |  |
| **GGT (gamma-glutamyl transferase)** | 47.6±17.92 | 63.7±45.75 | 57.4±36.68 | 60.2±27.70 | 0.617 | 55.3±26.55 | 41.8±18.82 | 59.8±38.26 | 63.0±35.32 | 0.425 |  |
| **HDL-C** | 46.1±11.26 | 42.7±4.74 | 44.2±8.67 | 47.4±13.43 | 0.639 | 45.3±11.22 | 51.3±12.98 | 43.9±8.80 | 43.7±9.28 | 0.289 |  |
| **LDL-C** | 134.6±35.13 | 132.6±23.18 | 122.5±35.78 | 134.8±28.51 | 0.703 | 130.8±39.73 | 134.3±19.20 | 136.9±34.66 | 126.8±27.22 | 0.808 |  |
| **Triglycerides** | 151.7±82.82 | 180.7±83.39 | 140.0±50.9 | 199.9±75.19 | 0.127 | 179.6±69.82 | 136.1±77.46 | 167.1±43.15 | 180.5±95.02 | 0.508 |  |
| **Total cholesterol** | 199.8±34.42 | 192.9±31.93 | 180.5±42.63 | 186.6±38.38 | 0.569 | 195.7±32.59 | 191.9±29.23 | 197.4±46.11 | 180.6±35.78 | 0.536 |  |
| **Fasting blood glucose** | 109.6±40.32 | 106.2±38.43 | 95.8±26.90 | 103.7±23.03 | 0.732 | 105.1±24.14 | 114.0±48.86 | 103.5±23.32 | 99.1±33.09 | 0.717 |  |
| **Fatty Liver Index (FLI)** | 72.0±29.11 | 75.0±19.68 | 76.2±17.56 | 77.5±19.07 | 0.915 | 83.2±17.992 | 62.3±30.83 | 75.1±16.92 | 76.4±19.88 | 0.170 |  |
| **Fatty Liver Index (FLI)** |  |  |  |  |  |  |  |  |  |  |  |
| ***<60*** | 3 (5.4) | 3 (5.4) | 2 (3.6) | 3 (5.4) | 0.932 | 1 (1.8) | 4 (7.1) | 2 (3.6) | 4 (7.1) | 0.192 |  |
| ***≥60*** | 11 (19.6) | 9 (16.1) | 11 (19.6) | 14 (25.0) |  | 11 (19.6) | 5 (8.9) | 12 (21.4) | 17 (30.4) |  |  |
| **Women** | | | | | | | | | | |  |
| **ALT (alanine transaminase)** | 34.8±16.56 | 45.9±28.32 | 43.7±25.14 | 30.8±14.71 | 0.234 | 40.8±28.42 | 39.7±20.43 | 42.2±24.05 | 32.1±14.44 | 0.787 |  |
| **AST (aspartate transaminase)** | 29.7±14.08 | 34.3±16.63 | 31.9±12.14 | 26.6±12.24 | 0.510 | 33.8±18.82 | 32.3±11.98 | 29.1±10.44 | 26.0±13.90 | 0.558 |  |
| **GGT (gamma-glutamyl transferase)** | 36.8±28.78 | 36.8±16.03 | 50.1±35.25 | 34.3±19.50 | 0.335 | 36.7±22.07 | 43.9±31.70 | 40.4±26.61 | 35.1±20.59 | 0.814 |  |
| **HDL-C** | 50.5±12.59 | 49.2±13.44 | 51.4±12.63 | 45.3±11.11 | 0.618 | 46.4±11.67 | 49.5±13.49 | 52.9±12.01 | 48.1±12.90 | 0.522 |  |
| **LDL-C** | 128.3±39.07 | 123.4±33.55 | 141.4±29.21 | 129.6±28.82 | 0.457 | 133.5±30.68 | 126.0±32.99 | 134.9±38.23 | 127.0±30.96 | 0.836 |  |
| **Triglycerides** | 175.5±89.05 | 141.6±56.48 | 171.8±79.09 | 142.3±65.24 | 0.418 | 189.9±87.60 | 136.2±53.41 | 159.9±72.45 | 140.9±72.15 | 0.140 |  |
| **Total cholesterol** | 188.3±54.23 | 188.1±43.50 | 209.0±42.48 | 187.8±33.83 | 0.462 | 195.2±43.93 | 193.2±43.06 | 189.8±50.63 | 198.5±43.38 | 0.973 |  |
| **Fasting blood glucose** | 108.1±14.13 | 117.0±40.63 | 126.6±42.75 | 104.9±22.77 | 0.306 | 111.4±19.00 | 117.4±38.97 | 120.5±42.79 | 105.3±25.8 | 0.716 |  |
| **Fatty Liver Index (FLI)** | 76.9±22.71 | 78.6±18.15 | 82.4±17.73 | 75.8±22.78 | 0.821 | 77.2±20.63 | 81.5±15.46 | 75.5±22.92 | 80.8±23.90 | 0.811 |  |
| **Fatty Liver Index (FLI)** |  |  |  |  |  |  |  |  |  |  |  |
| ***<60*** | 4 (6.6) | 2 (3.3) | 2 (3.3) | 3 (4.9) | 0.559 | 3 (4.9) | 2 (3.3) | 4 (6.6) | 2 (3.3) | 0.645 |  |
| ***≥60*** | 11 (18.0) | 16 (26.2) | 14 (23.0) | 9 (14.8) |  | 14 (23.0) | 18 (29.5) | 12 (19.7) | 6 (9.8) |  |  |

ANOVA, Kruskal Wallis, Chi-square
