# Supplementary material for: Are dietary factors associated with cardiometabolic risk factors in patients with non-alcoholic fatty liver disease?
Source: PeerJ. 2024 Jul 30;12:e17810. doi: 10.7717/peerj.17810 (PMC11296304; doi:10.7717/peerj.17810)
Supplement: Supplemental Information 5 — ANOVA, Kruskal Wallis, Chi-square [file peerj-12-17810-s005.docx]

**Supplementary Table 3.** Biochemical parameters of the participants in each quartile of DGI and DGL.

|  | ***DGI*** | | | | | ***DGL*** | | | | | |
| --- | --- | --- | --- | --- | --- | --- | --- | --- | --- | --- | --- |
|  | **Q1** | **Q2** | **Q3** | **Q4** | **p** | **Q1** | **Q2** | **Q3** | **Q4** | **p** |  |
| **Men** | | | | | | | | | | |  |
| **ALT (alanine transaminase)** | 59.9±45.54 | 53.0±24.32 | 65.1±29.91 | 61.8±57.64 | 0.912 | 49.3±13.30 | 48.6±22.90 | 68.8±38.71 | 66.3±56.17 | 0.495 |  |
| **AST (aspartate transaminase)** | 38.4±24.50 | 32.0±11.92 | 34.9±12.08 | 36.3±19.48 | 0.854 | 31.6±7.96 | 32.3±9.37 | 42.0±21.72 | 34.7±19.30 | 0.438 |  |
| **GGT (gamma-glutamyl transferase)** | 59.4±35.93 | 58.6±30.93 | 61.9±36.35 | 51.1±29.25 | 0.787 | 55.5±14.53 | 52.9±44.48 | 69.1±32.54 | 53.2±29.51 | 0.516 |  |
| **HDL-C** | 47.9±10.49 | 46.8±11.39 | 42.6±5.19 | 45.4±12.84 | 0.596 | 51.1±16.68 | 46.7±7.95 | 43.4±8.63 | 43.7±9.35 | 0.288 |  |
| **LDL-C** | 14..9±25.51 | 133.0±34.96 | 137.5±20.29 | 118.3±34.73 | 0.104 | 146.1±32.26 | 128.3±32.89 | 143.2±19.32 | 121.2±31.60 | 0.088 |  |
| **Triglycerides** | 214.8±118.84 | 137.8±68.30 | 172.3±57.99 | 162.6±59.40 | 0.131 | **187.5±68.11^a^** | **123.4±54.24^ab^** | **237.2±82.33^c^** | **149.9±59.42^ab^** | **0.000** |  |
| **Total cholesterol** | **222.3±24.08^a^** | **188.0±42.51^ab^** | **186.9±30.90^ab^** | **176.3±35.74^b^** | **0.006** | 214.6±37.52 | 187.5±44.84 | 198.9±29.31 | 177.3±32.22 | 0.063 |  |
| **Fasting blood glucose** | 113.3±47.52 | 97.1±15.27 | 107.5±36.88 | 99.8±24.63 | 0.607 | 125.9±49.08 | 99.7±16.98 | 102.3±30.32 | 99.3±30.33 | 0.212 |  |
| **Fatty Liver Index (FLI)** | 82.8±23.32 | 74.6±25.53 | 75.9±19.17 | 71.2±20.03 | 0.593 | 85.1±12.17 | 64.0±26.62 | 81.2±19.14 | 74.4±20.28 | 0.105 |  |
| **Fatty Liver Index (FLI)** |  |  |  |  |  |  |  |  |  |  |  |
| ***<60*** | 1 (1.8) | 1 (1.8) | 4 (7.1) | 5 (8.9) | 0.534 | 0 (0) | 4 (7.1) | 2 (3.6) | 5 (8.9) | 0.309 |  |
| ***≥60*** | 9 (16.1) | 10 (17.9) | 12 (21.4) | 14 (25.0) |  | 8 (14.3) | 8 (14.3) | 11 (19.6) | 18 (32.1) |  |  |
| **Women** | | | | | | | | | | |  |
| **ALT (alanine transaminase)** | 36.1±23.54 | 39.4±23.07 | 41.8±22.98 | 44.1±24.01 | 0.820 | 39.4±28.00 | 43.7±20.50 | 38.8±22.26 | 30.8±10.59 | 0.701 |  |
| **AST (aspartate transaminase)** | 28.9±15.19 | 29.2±11.46 | 31.5±13.10 | 38.1±16.88 | 0.350 | 32.2±18.26 | 33.0±10.79 | 30.1±12.38 | 23.3±8.73 | 0.506 |  |
| **GGT (gamma-glutamyl transferase)** | 33.3±22.31 | 40.9±23.84 | 46.2±30.85 | 41.8±31.86 | 0.575 | 37.0±21.65 | 47.2±31.30 | 39.5±29.04 | 28.5±10.60 | 0.435 |  |
| **HDL-C** | 49.8±12.51 | 50.7±12.50 | 48.0±13.95 | 47.7±11.90 | 0.908 | 51.0±13.27 | 46.1±11.16 | 50.2±12.78 | 51.0±13.90 | 0.634 |  |
| **LDL-C** | 126.4±41.39 | 134.4±26.68 | 125.9±36.64 | 137.2±21.77 | 0.759 | 135.3±33.66 | 127.5±33.18 | 129.6±35.22 | 125.5±30.14 | 0.868 |  |
| **Triglycerides** | 149.7±58.73 | 150.5±63.62 | 175.9±107.61 | 164.5±67.08 | 0.739 | 156.2±77.86 | 163.8±67.06 | 156.6±79.10 | 150.2±75.83 | 0.979 |  |
| **Total cholesterol** | 194.5±53.95 | 190.5±44.47 | 194.9±44.48 | 195.8±26.19 | 0.988 | 196.8±46.25 | 192.1±45.51 | 187.8±44.38 | 202.2±43.37 | 0.894 |  |
| **Fasting blood glucose** | 112.2±23.88 | 124.3±50.28 | 109.7±22.48 | 114.9±33.70 | 0.546 | 111.0±38.21 | 117.2±22.15 | 122.9±40.06 | 100.7±27.70 | 0.512 |  |
| **Fatty Liver Index (FLI)** | 76.9±21.82 | 78.4±19.46 | 77.7±23.42 | 83.6±12.19 | 0.855 | 74.5±20.30 | 81.7±16.14 | 78.6±23.21 | 84.0±20.87 | 0.634 |  |
| **Fatty Liver Index (FLI)** |  |  |  |  |  |  |  |  |  |  |  |
| ***<60*** | 4 (6.6) | 3 (4.9) | 4 (6.6) | 0 (0) | 0.283 | 4 (6.6) | 2 (3.3) | 4 (6.6) | 1 (1.6) | 0.770 |  |
| ***≥60*** | 15 (24.6) | 16 (26.2) | 9 (14.8) | 10 (16.4) |  | 17 (27.9) | 16 (26.2) | 12 (19.7) | 5 (8.2) |  |  |

ANOVA, Kruskal Wallis, Chi-square
