# Supplementary material for: Are dietary factors associated with cardiometabolic risk factors in patients with non-alcoholic fatty liver disease?
Source: PeerJ. 2024 Jul 30;12:e17810. doi: 10.7717/peerj.17810 (PMC11296304; doi:10.7717/peerj.17810)
Supplement: Supplemental Information 6 — ANOVA. Kruskal Wallis. Tukey’s Bold indicates statistically significant difference (p ≤ 0.05). Results with different letters (a-c) in the same row are significantly different. [file peerj-12-17810-s006.docx]

**Supplementary Table 4.** Participants' dietary energy and nutrient intake across quartiles of DII and DIL.

| **Nutrients** | ***DII*** | | | | | ***DIL*** | | | | |
| --- | --- | --- | --- | --- | --- | --- | --- | --- | --- | --- |
|  | **Q1** | **Q2** | **Q3** | **Q4** | **p** | **Q1** | **Q2** | **Q3** | **Q4** | **p** |
| **Men** | | | | | | | | | | |
| **Energy (kcal/d)** | 2335.6±1065.57 | 2097.6±610.96 | 2165.0±1115.46 | 1803.4±671.42 | 0.411 | **1680.8±968.45^a^** | **1721.6±635.52^a^** | **1764.0±443.71^a^** | **2681.6±870.34^b^** | **0.001** |
| **Protein (% Energy)** | 16.5±5.16 | 17.3±5.34 | 15.0±6.10 | 15.6±5.58 | 0.722 | 16.0±6.97 | 15.1±4.95 | 16.3±5.46 | 16.3±5.09 | 0.951 |
| **Total fat (% Energy)** | **34.2±11.60^ab^** | **41.7±6.89^a^** | **32.3±13.32^ab^** | **29.8±10.62^b^** | **0.042** | 33.7±15.07 | 40.2±13.93 | 32.5±10.97 | 32.7±7.83 | 0.382 |
| **Carbohydrate (% Energy)** | 42.9±11.82 | 40.3±10.77 | 42.2±14.29 | 48.2±9.13 | 0.271 | 38.0±10.77 | 39.3±12.18 | 45.6±11.34 | 47.8±10.72 | 0.061 |
| **Fiber (g/d)** | 27.8±19.86 | 22.2±10.14 | 29.5±20.10 | 19.3±8.39 | 0.242 | **23.7±22.29^ab^** | **14.7±5.60^a^** | **21.5±7.67^ab^** | **30.9±15.64^b^** | **0.046** |
| **Soluble fiber (g/d)** | 11.2±9.22 | 7.7±3.80 | 8.8±6.16 | 6.7±4.09 | 0.241 | **10.2±9.44^ab^** | **14.7±5.60^a^** | **21.5±7.67^ab^** | **30.9±15.64^b^** | **0.035** |
| **Insoluble fiber (g/d)** | 17.0±14.94 | 13.7±7.54 | 19.6±14.64 | 12.4±5.11 | 0.320 | 13.5±16.38 | 10.1±4.29 | 13.6±5.26 | 20.3±11.59 | 0.084 |
| **Fructose (g/d)** | 16.2±21.44 | 15.3±22.15 | 18.4±11.52 | 13.8±9.87 | 0.905 | 17.9±21.78 | 13.4±12.42 | 10.3±10.06 | 19.2±17.52 | 0.414 |
| **Sucrose (% Total Energy)** | 5.1±7.64 | 4.4±3.88 | 7.8±4.66 | 8.9±10.76 | 0.340 | 10.7±12.89 | 4.3±3.01 | 6.8±7.78 | 5.4±3.71 | 0.201 |
| **Saturated Fatty Acids (% Total Energy)** | 9.9±5.34 | 13.9±4.94 | 14.4±6.89 | 15.3±10.89 | 0.246 | 16.2±15.55 | 14.4±5.30 | 12.8±3.50 | 11.9±2.85 | 0.479 |
| **Mono-unsaturated Fatty Acids (% Total Energy)** | 9.7±4.72 | 16.0±6.47 | 15.1±8.37 | 12.6±8.64 | 0.132 | 15.9±14.85 | 13.3±3.44 | 12.4±4.02 | 12.2±3.53 | 0.564 |
| **Poly-unsaturated Fatty Acids (% Total Energy)** | 6.4±6.36 | 10.3±4.21 | 8.6±4.71 | 9.1±11.28 | 0.608 | 12.8±13.80 | 9.0±6.99 | 6.5±3.02 | 7.4±3.30 | 0.143 |
| **Cholesterol (mg/d)** | 336.2±231.82 | 373.2±171.46 | 371.1±160.51 | 311.5±215.72 | 0.809 | 299.1±243.44 | 415.1±238.95 | 273.9±139.79 | 388.0±169.18 | 0.200 |
| **n-6 : n-3** | **4.2±1.81^a^** | **17.3±10.94^b^** | **10.2±8.07^ab^** | **8.6±9.91^ac^** | **0.003** | 7.4±11.59 | 7.5±6.08 | 10.3±9.49 | 11.7±9.21 | 0.524 |
| **Women** | | | | | | | | | | |
| **Energy (kcal/d)** | 1640.3±835.22 | 1446.7±432.72 | 1518.9±623.58 | 1685.9±505.20 | 0.698 | **1467.8±916.12^a^** | **1332.2±346.75^a^** | **1667.8±336.22^a^** | **2112.3±361.94^b^** | **0.013** |
| **Protein (% Energy)** | 18.1±7.00 | 16.4±4.71 | 15.8±4.40 | 13.5±2.71 | 0.144 | 16.2±7.53 | 17.5±4.84 | 15.3±2.96 | 14.0±1.24 | 0.350 |
| **Total fat (% Energy)** | 36.8±12.10 | 38.0±10.39 | 32.2±10.32 | 32.0±7.68 | 0.259 | 35.7±12.62 | 36.4±10.96 | 34.6±8.94 | 30.7±7.08 | 0.631 |
| **Carbohydrate (% Energy)** | **40.8±11.20^a^** | **42.6±10.12^ab^** | **50.9±10.36^b^** | **52.5±9.47^b^** | **0.006** | 42.7±15.61 | 43.5±6.89 | 49.7±9.64 | 54.4±6.59 | 0.062 |
| **Fiber (g/d)** | 24.6±8.70 | 20.4±9.61 | 22.9±9.17 | 21.1±12.23 | 0.628 | 19.7±9.61 | 20.3±8.83 | 25.2±9.28 | 26.3±11.97 | 0.188 |
| **Soluble fiber (g/d)** | 8.5±4.84 | 6.2±4.31 | 7.3±5.12 | 6.2±3.46 | 0.438 | 8.1±7.03 | 5.9±2.83 | 7.1±3.29 | 7.6±3.16 | 0.500 |
| **Insoluble fiber (g/d)** | 15.7±6.60 | 13.1±7.12 | 13.9±5.89 | 14.9±9.14 | 0.753 | 11.1±4.60 | 13.9±6.51 | 16.2±7.67 | 18.5±9.00 | 0.059 |
| **Fructose (g/d)** | 10.6±5.67 | 11.7±8.96 | 13.0±9.51 | 15.4±11.41 | 0.560 | 10.2±4.55 | 10.1±6.54 | 15.1±12.06 | 18.0±11.33 | 0.072 |
| **Sucrose (% Total Energy)** | **5.3±3.16^a^** | **5.3±2.91^a^** | **5.2±3.01^a^** | **10.7±6.08^b^** | **0.001** | **3.8±2.34^a^** | **5.7±2.77^a^** | **7.4±5.03^b^** | **11.2±5.07^bc^** | **0.000** |
| **Saturated Fatty Acids (% Total Energy)** | 11.6±5.10 | 13.6±4.82 | 12.5±3.24 | 12.5±5.51 | 0.685 | 11.7±5.82 | 13.8±3.80 | 12.5±4.10 | 12.0±4.95 | 0.570 |
| **Mono-unsaturated Fatty Acids (% Total Energy)** | 14.4±6.43 | 14.0±4.12 | 12.6±3.57 | 11.2±2.72 | 0.219 | 12.9±6.43 | 14.8±3.60 | 12.8±3.39 | 10.6±2.43 | 0.150 |
| **Poly-unsaturated Fatty Acids (% Total Energy)** | 8.0±5.22 | 7.9±3.85 | 6.1±2.45 | 6.8±3.05 | 0.441 | 7.2±6.43 | 7.4±2.61 | 7.4±4.46 | 6.3±1.88 | 0.915 |
| **Cholesterol (mg/d)** | 223.4±143.93 | 222.1±122.62 | 286.1±210.40 | 178.3±145.87 | 0.354 | 212.8±194.64 | 224.3±111.20 | 259.1±182.24 | 227.0±155.58 | 0.866 |
| **n-6 : n-3** | 10.3±8.41 | 9.1±7.05 | 9.1±6.38 | 10.3±9.93 | 0.953 | 7.3±8.32 | 10.2±6.99 | 11.5±8.07 | 9.5±7.42 | 0.468 |

ANOVA. Kruskal Wallis. Tukey’s

Bold indicates statistically significant difference (*P*≤0.05). Results with different letters (a-c) in the same row are significantly different.
