# Supplementary material for: Are dietary factors associated with cardiometabolic risk factors in patients with non-alcoholic fatty liver disease?
Source: PeerJ. 2024 Jul 30;12:e17810. doi: 10.7717/peerj.17810 (PMC11296304; doi:10.7717/peerj.17810)
Supplement: Supplemental Information 7 — ANOVA. Kruskal Wallis. Tukey’s Bold indicates statistically significant difference (p ≤ 0.05). Results with different letters (a-c) in the same row are significantly different. [file peerj-12-17810-s007.docx]

**Supplementary Table 5.** Participants' dietary energy and nutrient intake across quartiles of DGI and DGL.

| **Nutrients** | ***DGI*** | | | | | ***DGL*** | | | | | |
| --- | --- | --- | --- | --- | --- | --- | --- | --- | --- | --- | --- |
|  | **Q1** | **Q2** | **Q3** | **Q4** | **p** | **Q1** | **Q2** | **Q3** | **Q4** | **p** |  |
| **Men** | | | | | | | | | | |  |
| **Energy (kcal/d)** | 1725.6±620.60 | 2383.4±972.84 | 2203.2±1030.03 | 1997.3±810.94 | 0.347 | 1701.7±1027.58 | 1895.2±496.73 | 1769.5±806.63 | 2491.9±921.94 | 0.075 |  |
| **Protein (% Energy)** | 19.0±7.03 | 17.6±4.85 | 15.6±2.86 | 14.1±6.05 | 0.099 | 18.6±6.30 | 14.7±3.72 | 17.2±6.89 | 15.3±4.97 | 0.328 |  |
| **Total fat (% Energy)** | 35.7±15.00 | 35.4±5.56 | 36.2±11.35 | 30.7±12.13 | 0.474 | 37.0±14.59 | 37.6±11.94 | 35.5±12.65 | 30.4±8.82 | 0.236 |  |
| **Carbohydrate (% Energy)** | **35.5±13.03^a^** | **47.0±8.17^ab^** | **47.7±12.76^b^** | **43.0±9.78^ab^** | **0.045** | 39.0±11.45 | 38.8±11.70 | 45.6±11.53 | 47.0±10.91 | 0.124 |  |
| **Fiber (g/d)** | 19.5±8.79 | 24.6±10.84 | 31.4±23.34 | 21.0±10.47 | 0.162 | 16.9±12.60 | 22.0±6.49 | 24.4±20.36 | 28.3±16.28 | 0.315 |  |
| **Soluble fiber (g/d)** | 6.7±3.85 | 7.9±4.79 | 10.8±6.80 | 7.8±7.33 | 0.350 | 8.5±10.62 | 6.8±2.95 | 7.6±5.69 | 9.9±6.00 | 0.539 |  |
| **Insoluble fiber (g/d)** | 12.2±6.31 | 16.0±6.67 | 20.5±18.11 | 12.8±5.80 | 0.165 | 8.0±4.23 | 14.5±4.77 | 16.4±15.25 | 18.2±11.99 | 0.169 |  |
| **Fructose (g/d)** | 16.6±12.39 | 11.8±10.30 | 17.2±19.50 | 16.4±18.72 | 0.847 | 9.6±10.46 | 9.7±10.19 | 13.5±8.66 | 22.4±21.37 | 0.073 |  |
| **Sucrose (% Total Energy)** | 3.9±3.57 | 4.6±3.25 | 6.5±7.49 | 9.6±10.26 | 0.188 | **3.1±2.37^ab^** | **2.9±1.44^a^** | **11.5±10.24^b^** | **7.3±7.79^ab^** | **0.016** |  |
| **Saturated Fatty Acids (% Total Energy)** | 12.0±7.12 | 11.9±3.06 | 14.5±3.62 | 14.2±10.90 | 0.751 | 11.5±7.54 | 13.1±4.39 | 17.5±11.48 | 12.0±6.30 | 0.182 |  |
| **Mono-unsaturated Fatty Acids (% Total Energy)** | 10.5±5.34 | 11.7±2.66 | 13.9±7.73 | 14.9±9.76 | 0.429 | 10.5±5.52 | 12.7±4.17 | 17.3±11.59 | 12.1±5.96 | 0.146 |  |
| **Poly-unsaturated Fatty Acids (% Total Energy)** | 5.8±6.94 | 9.0±3.72 | 8.0±4.62 | 10.3±10.80 | 0.487 | 6.3±7.58 | 7.6±3.99 | 10.6±11.02 | 8.7±6.70 | 0.600 |  |
| **Cholesterol (mg/d)** | 316.9±212.71 | 346.3±239.26 | 332.2±144.24 | 369.1±210.95 | 0.911 | 228.0±194.56 | 350.6±222.97 | 302.0±158.87 | 406.4±188.15 | 0.122 |  |
| **n-6 : n-3** | 5.1±3.85 | 12.7±10.76 | 11.2±8.48 | 9.3±10.84 | 0.264 | 3.5±1.76 | 12.2±9.43 | 8.2±6.45 | 11.5±11.38 | 0.133 |  |
| **Women** | | | | | | | | | | |  |
| **Energy (kcal/d)** | **1228.6±396.89^a^** | **1496.6±496.98^abc^** | **1888.2±712.29^b^** | **1885.2±678.57^c^** | **0.004** | **1070.4±307.50^a^** | **1760.3±675.46^b^** | **1718.6±447.81^b^** | **2252.8±297.13^b^** | **0.000** |  |
| **Protein (% Energy)** | **18.4±5.54^a^** | **17.9±4.19^a^** | **13.3±3.63^b^** | **11.8±3.29^b^** | **0.000** | **18.4±5.64^a^** | **16.1±5.51^ab^** | **13.7±3.66^b^** | **14.3±1.03^ab^** | **0.035** |  |
| **Total fat (% Energy)** | 38.9±8.38 | 36.1±11.68 | 30.3±10.62 | 31.4±9.29 | 0.082 | **39.7±7.56^a^** | **34.8±14.17^ab^** | **30.8±7.43^b^** | **30.5±8.07^ab^** | **0.044** |  |
| **Carbohydrate (% Energy)** | **42.1±11.39^a^** | **43.4±9.04^a^** | **56.2±8.88^b^** | **47.1±10.87^ab^** | **0.002** | **40.7±10.95^ab^** | **42.8±8.71^a^** | **54.7±9.02^b^** | **54.5±7.40^b^** | **0.000** |  |
| **Fiber (g/d)** | 22.8±10.93 | 20.6±7.47 | 23.1±9.75 | 22.9±12.22 | 0.868 | 18.1±8.12 | 23.2±7.35 | 24.7±12.06 | 26.9±11.76 | 0.094 |  |
| **Soluble fiber (g/d)** | 6.9±3.38 | 5.1±2.18 | 8.4±5.92 | 9.4±6.38 | 0.063 | 5.8±4.00 | 7.5±5.34 | 7.7±4.63 | 8.4±3.01 | 0.483 |  |
| **Insoluble fiber (g/d)** | 15.4±7.92 | 13.4±6.18 | 14.5±5.58 | 13.9±9.07 | 0.847 | 11.8±5.77 | 13.7±4.90 | 16.7±8.92 | 18.9±8.34 | 0.061 |  |
| **Fructose (g/d)** | 13.7±7.43 | 10.2±8.86 | 16.7±11.74 | 8.9±4.93 | 0.100 | **11.1±5.84^ab^** | **9.0±6.03^a^** | **15.2±11.74^ab^** | **20.5±11.25^b^** | **0.017** |  |
| **Sucrose (% Total Energy)** | 5.0±2.52 | 5.7±2.61 | 8.2±5.43 | 7.7±6.86 | 0.143 | **4.7±2.20^a^** | **5.0±3.07^ab^** | **8.5±5.54^b^** | **10.1±5.47^c^** | **0.002** |  |
| **Saturated Fatty Acids (% Total Energy)** | 14.3±4.52 | 13.0±3.32 | 11.6±5.66 | 9.8±4.54 | 0.070 | 14.0±4.55 | 12.9±4.50 | 10.8±4.23 | 11.7±5.56 | 0.190 |  |
| **Mono-unsaturated Fatty Acids (% Total Energy)** | **14.3±4.12^a^** | **15.3±4.17^a^** | **10.1±3.11^b^** | **11.2±4.93^ab^** | **0.002** | **15.2±4.15^a^** | **14.1±5.43^ab^** | **10.5±2.35^b^** | **10.5±2.74^ab^** | **0.003** |  |
| **Poly-unsaturated Fatty Acids (% Total Energy)** | 8.1±4.56 | 7.6±3.90 | 5.3±2.26 | 7.4±3.21 | 0.209 | 8.0±4.35 | 7.2±4.07 | 6.4±3.26 | 6.6±2.11 | 0.659 |  |
| **Cholesterol (mg/d)** | 171.5±134.48 | 261.6±157.83 | 302.0±190.21 | 191.1±130.55 | 0.085 | 200.3±130.45 | 221.2±118.07 | 285.7±228.86 | 217.6±137.45 | 0.438 |  |
| **n-6 : n-3** | 10.7±8.71 | 8.2±5.65 | 9.1±6.15 | 11.1±10.91 | 0.702 | 9.4±8.67 | 8.9±6.96 | 11.2±7.88 | 8.1±6.79 | 0.790 |  |

ANOVA. Kruskal Wallis. Tukey’s

Bold indicates statistically significant difference (*P*≤0.05). Results with different letters (a-c) in the same row are significantly different.
